# Different approaches to long-term treatment of aHUS due to *MCP* mutations: a multicenter analysis

**DOI:** 10.1007/s00467-020-04714-0

**Published:** 2020-07-26

**Authors:** Verena Klämbt, Charlotte Gimpel, Martin Bald, Christopher Gerken, Heiko Billing, Sebastian Loos, Matthias Hansen, Jens König, Tobias Vinke, Carmen Montoya, Bärbel Lange-Sperandio, Martin Kirschstein, Imke Hennies, Martin Pohl, Karsten Häffner

**Affiliations:** 1 Department of General Pediatrics, Adolescent Medicine and Neonatology, Medical Center, University of Freiburg, Faculty of Medicine, Mathildenstr. 1, 79106 Freiburg, Germany; 2 Department of Internal Medicine IV, Medical Center, University of Freiburg, Faculty of Medicine, Freiburg, Germany; 3 Olga Children’s Hospital, Department of Pediatric Nephrology, Klinikum Stuttgart, Stuttgart, Germany; 4 University Children’s Hospital Marburg, Philipps-University, Marburg, Germany; 5 Paediatrics I, University Children’s Hospital, Tübingen, Germany; 6 University Children’s Hospital, University Medical Center Hamburg-Eppendorf, Hamburg, Germany; 7 KfH-Nierenzentrum für Kinder und Jugendliche beim Clementine-Kinderhospital, Frankfurt, Germany; 8 Department of General Pediatrics, University Children’s Hospital, Münster, Germany; 9 Department of Pediatrics I, University Children’s Hospital of Heidelberg, Heidelberg, Germany; 10 KfH Center of Pediatric Nephrology, Children’s Hospital Munich Schwabing, Munich, Germany; 11 Dr. v. Hauner Children’s Hospital, Ludwig Maximilians University, Munich, Germany; 12 Department of Pediatrics, General Hospital, Celle, Germany; 13 Department of Paediatric Kidney, Liver and Metabolic Diseases, Paediatric Research Center, Hannover Medical School Children’s Hospital, Hannover, Germany

**Keywords:** Atypical hemolytic uremic syndrome, Discontinuation, Genetic cause, Membrane cofactor protein, Complement, Eculizumab, Children

## Abstract

**Background:**

Atypical hemolytic uremic syndrome (aHUS) is a rare, life-threatening microangiopathy, frequently causing kidney failure. Inhibition of the terminal complement complex with eculizumab is the only licensed treatment but mostly requires long-term administration and risks severe side effects. The underlying genetic cause of aHUS is thought to influence the severity of initial and recurring episodes, with milder courses in patients with mutations in *membrane cofactor protein* (*MCP*).

**Methods:**

Twenty pediatric cases of aHUS due to isolated heterozygous *MCP* mutations were reported from 12 German pediatric nephrology centers to describe initial presentation, timing of relapses, treatment, and kidney outcome.

**Results:**

The median age of onset was 4.6 years, with a female to male ratio of 1:3. Without eculizumab maintenance therapy, 50% (9/18) of the patients experienced a first relapse after a median period of 3.8 years. Kaplan-Meier analysis showed a relapse-free survival of 93% at 1 year. Four patients received eculizumab long-term treatment, while 3 patients received short courses. We could not show a benefit from complement blockade therapy on long term kidney function, independent of short-term or long-term treatment. To prevent 1 relapse with eculizumab, the theoretical number-needed-to-treat (NNT) was 15 for the first year and 3 for the first 5 years after initial presentation.

**Conclusion:**

Our study shows that heterozygous *MCP* mutations cause aHUS with a risk of first relapse of about 10% per year, resulting in large NNTs for prevention of relapses with eculizumab. More studies are needed to define an optimal treatment schedule for patients with *MCP* mutations to minimize the risks of the disease and treatment.

**Electronic supplementary material:**

The online version of this article (10.1007/s00467-020-04714-0) contains supplementary material, which is available to authorized users.

## Introduction

Atypical hemolytic uremic syndrome (aHUS) is a very rare, life-threatening thrombotic microangiopathy mainly caused by uncontrolled complement activation with a reported incidence of 0.5 per million per year [1]. Approximately, 50–70% of the patients have causative mutations in genes encoding complement factor H (20–30%), membrane cofactor protein (*MCP*; 10–15%), complement factor I (4–10%), factor B (1–2%), C3 (5–10%), thrombomodulin (5%), or diacylglycerol kinase ɛ (3%), while 6% of aHUS patients have anti-factor H antibodies [2–4]. The underlying genetic or immunologic cause is thought to determine the clinical outcome. While patients with mutations in complement factor H suffer mostly from a severe clinical course, patients with an isolated *MCP* mutation have a more favorable outcome despite reported relapses in up to 90% [5–7]. Approximately, 80% of the pediatric patients with *MCP* mutations show no long-term kidney impairment [5, 7]. In an Italian cohort of MCP-aHUS children, none of the patients (0/13) progressed to chronic kidney disease (CKD) stage 5 or had CKD with proteinuria at 1- or 3-year follow-up [7, 8]. In a French series with longer follow-up periods, 0% (0/12) of the children with MCP-aHUS progressed to CKD stage 5 at 1-year follow-up, while 17% (2/12) or 25% (3/12) showed CKD stage 5 at 5 years or median last follow-up of 17.8 years, respectively [5].

Prior to the availability of effective treatment, aHUS patients had a very poor prognosis as up to 78% of the patients died or developed kidney failure within a few years [3, 5, 7–10]. Treatment options were limited to plasma exchange or plasma infusion therapy. Since 2011, eculizumab, a monoclonal antibody targeting complement factor C5, has revolutionized the management of aHUS patients [11, 12]. Eculizumab binds to the C5 protein thereby blocking its cleavage into the proinflammatory C5a and the pro-thrombotic C5b, which is a precursor of the lytic terminal complement complex. It is highly effective for treating acute episodes and preventing relapses, but needs to be administered intravenously at frequent intervals for maintenance therapy [11]. In 2016, an international consensus approach for the management of aHUS in children recommended long-term eculizumab treatment for aHUS patients with a good response to treatment independently of the underlying mutation [3].

Over the last years, discontinuation of long-term eculizumab treatment has been discussed controversially, but observational studies and guidelines are missing [13–18]. The main rationale for discontinuation of eculizumab is to reduce the risk of severe side effects and high treatment costs. Side effects include the higher risk for meningococcal infections and complications due to intravenous injections and potential long-term immune-mediated drug reactions, even though this has so far not been observed [19–21]. Furthermore, infusions twice a month have a high impact on the patients’ quality of life. Therefore, discontinuation of the recommended therapy is considered by many families and pediatric nephrologists, especially for children with *MCP* mutations who have a reported low overall risk of kidney failure.

Here, we describe the initial aHUS presentation, timing of relapses, treatment, and kidney outcome of 20 children from German pediatric nephrology centers with aHUS due to isolated heterozygous *MCP* mutations. The objective of the present retrospective study was to report on the clinical presentation and to compare the outcome of patients receiving or not receiving eculizumab.

## Methods

### Study design

The study used an anonymized questionnaire that was sent to all members of the German Society for Pediatric Nephrology (Gesellschaft für Pädiatrische Nephrologie (GPN)). Twenty patients from 12 different German centers were enrolled in this study (Table 1, Suppl. Table 1). Only patients with an isolated *MCP* mutation were included. Genetic testing was performed by the individual hospitals, and variants were reported. The significance of different missense variants was ranked based on their predicted impact on protein sequence and function considering evolutionary conservation among orthologs across phylogeny, and web-based pathogenicity prediction programs (PolyPhen-2 [22], SIFT [23] and MutationTaster [24]). Deleteriousness was assessed using established criteria based on ACMG [25]. The Human Gene Mutation Database Professional (https://portal.biobase-international.com/hgmd/pro/start.php) was used to check whether mutations had been previously reported. All cases of aHUS that had other genetic causes or patients with HUS secondary to infections, autoimmune diseases, drugs, organ transplantation, pregnancy, or cobalamin deficiency were not included in this study. Questions included age of onset, sex, *MCP* genotype, severity of onset with maximum serum creatinine, days of hospitalization, and extra-renal manifestations. The treatment modalities including days on dialysis, transfusions, plasma exchange, and eculizumab were recorded as well as the outcome after the first episode. A similar questionnaire was completed for every relapse. The estimated glomerular filtration rate (eGFR) was calculated based on the Pottel formula [26, 27].

**Table 1:** Clinical information on 20 patients with heterozygous MCP mutation and aHUS

Family Nr.	Gender	Age of onset (yrs)	Age at first relapse (yrs)	Time until first relapse (yrs)	Nr. of relapses	Best GFR after FM	Best GFR last follow-up	Years of follow-up	Eculizumab
1	m	1.6	6.8	5.3	2	148	5	7.8	/
2	m	15.3	/	/	0	98	98	0.2	/
3	m	5.8	7.6	1.8	1	70	118	1.8	/
4	m	1.9	2.7	0.7	11	102	105	14.8	Long-term after the eleventh relapse
5	f	4.4	/	/	0	148	148	2.3	/
6	m	0.6	2.3	1.8	1	67	82	2.3	Long-term after FM
7	f	4.8	/	/	0	108	105	0.3	/
8	m	8	14	6	1	69	94	6	/
9	m	1.2	/	/	0	114	125	2.8	/
10	m	14.1	/	/	0	97	85	2.4	/
11	f	2.8	/	/	0	144	112	2.3	Long-term after FM
12	f	6	/	/	0	150	151	6.2	/
13	m	3	6.8	3.8	3	113	124	9.8	Long-term after the third relapse
14	m	3.2	10.7	7.5	2	63	85	13.8	/
15	m	12.3	/	/	0	80	80	0.5	/
16	m	4.8	14.3	9.4	1	70	82	9.5	After the first relapse, on-demand
17	m	4.1	5.8	1.6	2	151	133	3.3	After the first relapse, on-demand
18	m	10.7	/	/	0	108	124	1.8	After FM, on-demand
19	f	4.1	/	/	0	96	90	1.4	/
20	m	6.1	7.5	1.4	7	69	93	15.9	/

*GFR* glomerular filtration rate, *f* female, *FM* first manifestation, *m* male, *Nr* number, *Y* yes, *yrs* years. GFR in ml/min/1.73 m^2^

/, not applicable

The diagnosis of first presentation and relapse of HUS was made on the grounds of clinical criteria (microangiopathic hemolytic anemia, thrombocytopenia, and acute kidney injury) by the treating physicians. Throughout the manuscript, we define long-term eculizumab treatment as infusions twice a month with doses adjusted to body weight as currently recommended [3]. If eculizumab was only administered during initial aHUS and/or relapse episodes, we define this a short-term treatment as specified by the treating physicians. The study was approved by the local ethics committee.

### Statistics

Data were stored and analyzed using Excel, GraphPad Prism 8 (San Diego, CA), and SAS V9.4 (Cary, NJ). Descriptive statistics are given as median and interquartile range (IQR, 25th to 75th percentile). To avoid assuming normal distribution of continuous variables, non-parametric tests were used throughout. For comparing patients with vs. without relapse, continuous variables were tested using the Wilcoxon signed-rank test and categorical variables using Fisher’s exact test. *p* values of less than 0.05 were considered significant. Kaplan-Meier analysis of relapse-free survival was performed using SAS Proc Lifetest. From this, the maximum absolute risk reduction of suffering a relapse achievable through long-term eculizumab treatment was calculated as 1 - survival. This assumes the “best case scenario” that all relapses can be prevented with eculizumab, which is reasonable given the data of clinical trials [3, 11, 12]. The number of patients needed to treat (NNT) with eculizumab to prevent 1 relapse was calculated as 1/absolute risk reduction. For the analysis of kidney outcome, the patient who progressed to CKD stage 5 was censored just before kidney transplantation with an eGFR of 5 ml/min × 1.73m^2^.

## Results

### Patient characteristics and initial presentation

Twenty aHUS patients from 20 unrelated families with isolated *MCP* mutations from 12 different German pediatric nephrology centers were included (Table 1, Suppl. Table 1). All patients had a heterozygous *MCP* mutation, 12 of these had been previously reported (Suppl. Table 1) [28–31]. The female-to-male ratio among the patients was 1:3, with 5 girls and 15 boys (Table 2). The median age of the first aHUS episode was 4.6 years (IQR 2.8–7.5 years), and 11 patients had an upper respiratory tract infection or gastroenteritis as an identified trigger (Table 2). Kidney function decreased to a median eGFR of 22 ml/min/1.73m^2^ (IQR: 15–41 ml/min/1.73m^2^) during the first episode. Nine patients required transfusions due to severe anemia or thrombocytopenia. None of the patients received dialysis. The median hospitalization time was 9 days, and 4 patients were treated with plasmapheresis or plasma therapy. Three patients received eculizumab during the first episode, and two of these patients continued treatment as long-term therapy (Table 1 and 2). The median eGFR recovered to 100 ml/min/1.73m^2^ (IQR: 70–114 ml/min/1.73m^2^) after the initial episode. There were no reported extrarenal manifestations.

**Table 2:** Characteristics of patient cohort during initial aHUS presentation, number of relapses, and renal outcome

	All patients *N* = 20	Patients without long-term eculizumab *N* = 18		
		No relapse *N* = 9	Relapse *N* = 9	No-relapse vs. relapse *p* value
Gender (male/female)	15/5	5/4	9/0	0.04
Age at the first episode (years)	4.58 (2.8–7.5)	6 (4–13.1)	4.1 (2.5–6.0)	0.054
Follow-up time (years)	2.6 (1.8–9.1)	1.7 (0.4–2.6)	9.5 (4.6–14.3)	< 0.001
Age at last follow-up (years)	12.29 (5.8–15.21)	12.2 (5.3–14.1)	14.0 (8.5–16.8)	0.15
Time until first relapse Age at the first relapse (years)	-	-	3.75 (1.5–6.7) 7.5 (6.2–12.3)	-
Number of relapses				-
0	10	9	0	
1	3	0	2	
2	4	0	4	
3–6	1	0	1	
7–11	2	0	2	
GFR (ml/min/1.73 m^2^)				
Lowest in the first episode	22 (15–41)	22 (10–34)	39 (16–46)	0.13
Best after the first episode	100 (70–114)	106 (96–111)	70 (69–131)	0.50
Lowest in the first relapse	-	-	23 (15–41)	-
Best after the first relapse	-	-	94 (80–120)	-
At last follow-up	102 (85–124)	105 (81–136)	94 (84–121)	0.25
Characteristics of the first episode				
Received erythrocytes or platelets	9	4	5	-
Received plasma therapy/plasmapheresis	4	2	1	-
Received short-term eculizumab (overall)	1 (3)	1	0 (2)	-
Received long-term eculizumab (overall)	2 (4)	0	0 (2)	-
Days in hospital	9.0 (7.2–14.5)	9.0 (7.5–12)	8.0 (6.0–13.0)	0.38

Values are shown as *n* or median (interquartile range)

*p* values for two-sided Wilcoxon rank sum tests for continuous variables and Fisher’s exact test for categorical variables

*n.a.* not applicable

### Relapses

In total, 10 of the 20 patients (50%) experienced at least one relapse, including patient number 6, who had received long-term eculizumab treatment, but missed 5 administrations prior to relapse. To avoid bias, only the 18 patients without initial long-term eculizumab were considered for the analysis of relapse frequencies from here on. Of these, 9 patients experienced a first relapse after a median time of 3.8 years (IQR: 1.5–6.7) at a median age of 7.5 years (IQR: 6.2–12.3 years) (Table 2).

**Fig. 1:**
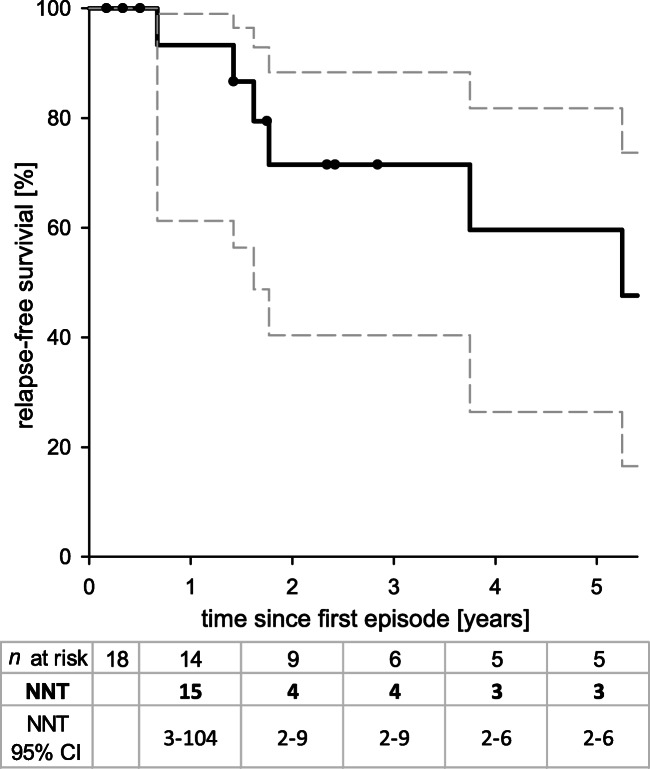
Kaplan-Meier analysis of survival without relapse after the end of the presenting episode of atypical hemolytic uremic syndrome in 18 children with *MCP* mutations without long-term eculizumab treatment. NNT: number needed to treat (i.e., this number of children would need to be treated for the whole corresponding time span to prevent 1 first relapse, assuming eculizumab could have prevented all relapses). 95% CI: 95% confidence interval

Because the median follow-up time of patients with a relapse was significantly longer than that of the non-relapsing group (9.5 years vs. 1.7 years, *p* < 0.01, Table 2), we performed Kaplan-Meier survival analysis to estimate relapse likelihood. The Kaplan-Meier plot in Fig. 1 shows that 7%, 29%, and 40% of children had suffered a first relapse after 1, 3, and 5 years respectively without long-term treatment. The risk of a first relapse was approximately 10% per year over the entire study period. Relapses were associated with viral infections of the upper airways or infections of the gastrointestinal tract in 4 out of 10 cases. During the first relapse, median eGFR decreased to 23 ml/min/1.73m^2^ (IQR: 15–41 ml/min/1.73m^2^). The median hospitalization time was 10 days (IQR: 8.5–15 days), 3 patients required dialysis and 5 patients received blood transfusions. Two patients were treated with plasmapheresis and/or plasma therapy and 2 patients with eculizumab. The median eGFR after the first relapse was 94 ml/min/1.73m^2^ (IQR: 80–120 ml/min/1.73m^2^).

Of note, all patients experiencing relapses were male and tended to be younger compared with those without relapsing disease (4.1 vs. 6 years, *p* = 0.054, Table 2). Seven patients experienced more than one relapse episode (2 relapses *n* = 4; 3 or more relapses *n* = 3; Table 2).

### Eculizumab treatment

Two patients received eculizumab long-term treatment starting from the first aHUS presentation (patient 6 and 11). Both patients received a drug dose according to current recommendations adjusted to body weight. Patient 6 presented at the age of 7 months and his eGFR only recovered to 67 ml/min/1.73m^2^ after the first episode despite eculizumab therapy. After receiving continuous treatment, the patient missed eculizumab administrations for 2.5 months and relapsed at the age of 2.33 years. Treatment was resumed and kidney function increased to an eGFR of 82 ml/min/1.73m^2^. Patient 11 initially presented with aHUS at the age of 2.75 years. The patient’s best eGFR was 144 ml/min/1.73m^2^ after the first episode (17 ml/min/1.73m^2^ during the first episode) and remained normal at last follow-up. Furthermore, 2 patients received eculizumab as long-term treatment after the eleventh or third relapse (patient 4 and 13). Both patients had normal kidney function at the time of last follow-up (eGFR 104 and 125 ml/min/1.73m^2^). At last follow-up, no further relapses had occurred in these patients.

Assuming that eculizumab could have prevented every relapse that occurred in patients without long-term treatment, the number needed to treat (NNT) to prevent 1 relapse was calculated: for the first year after initial presentation, the NNT was 15 with a wide 95% confidence interval of 3–104. For the whole 5-year period, the NNT was 3 (95% confidence interval 2–6).

Three additional patients (16, 17, and 18) only received short-term eculizumab therapy (3–5 eculizumab infusions). Patient 18 received this treatment already during the first aHUS episode, while patients 16 and 17 received short-term eculizumab courses after the first relapse (Table 1). Apart from patient 16, who had an eGFR at last follow-up of 82 ml/min/1.73m^2^, the other patients had a reported eGFR above 90 ml/min/1.73m^2^.

### Kidney outcomes

The reported maximum eGFR directly after the first aHUS episode did not differ significantly in patients with vs. without relapse excluding the 2 patients with long-term eculizumab treatment (Table 2). The non-relapsing group had an increase of median lowest eGFR from 22 to 106 ml/min/1.73m^2^, while the relapsing group had a reported eGFR rise from 39 to 70 ml/min/1.73m^2^ (*p* = ns, Table 2). The median eGFR at last follow-up was again similar in both groups (94 vs. 105 ml/min/1.73m^2^, Table 2).

The overall median kidney function of all 20 patients at last follow-up was 102 ml/min/1.73m^2^. In total, 14 out of 20 patients had an eGFR over 90 ml/min/1.73m^2^ (Table 1), while 5 patients developed CKD stage 2, and 1 patient CKD stage 5 at last follow-up (Table 1).

One patient (patient 1) required chronic dialysis 5.58 years after the first onset of disease, shortly after the second relapse. He never received eculizumab. The patient received a kidney transplant at the age of 9 years, 7.8 years after initial presentation and no relapse had occurred 9 years after transplantation.

The 7 patients who received either short- or long-term eculizumab had a slightly, but not significantly, better kidney function than the 13 patients who did not receive eculizumab (112 (IQR: 82–124) vs. 94 (IQR: 85–122) ml/min/1.73m^2^, *p* = 0.58). There was no significant difference in kidney function at last follow-up between the groups receiving short-term eculizumab vs. long-term eculizumab; however, due to small case numbers, this should be interpreted with care (124 (IQR: 82–133) vs. 108.3 (87–121) ml/min/1.73m^2^, *p* = 0.67).

## Discussion

Here, we describe one of the largest pediatric cohorts of patients suffering from aHUS due to isolated *MCP* mutations. Most aHUS studies have included both adult and pediatric patients and have analyzed mutations in a variety of genes together [32, 33]. However, it appears likely that different underlying genetic causes have variable disease outcomes. Furthermore, a previous study found that pediatric patients with aHUS due to *MCP* mutations have a more favorable long-term kidney outcome compared with adult patients [5].

In contrast to previous data [5, 34, 35], males were predominant in our cohort. This could be explained by the fact that other groups have reported on patients with all genetic mutations, not focusing on patients with isolated *MCP* mutation. However, larger case studies are needed to validate this finding. The mean age of onset in our cohort (4.6 years) was comparable with that reported in the cohort of the global (3.8 years) [35] and Turkish (4.8 years) [36] aHUS registries published in 2018, but higher compared with the mean age of onset published by the French registry in 2013 (1.5 years) [5].

Despite the fact that kidney function appears to be well preserved in children with *MCP* mutations, variable relapse rates of 20 [7] up to 92% [5] after a median follow-up time of 3 and 17.8 years, respectively, have been described. In our cohort, Kaplan-Meier analysis showed a relapse-risk rate of 7%, 29%, and 40% at 1, 3, and 5 years after first manifestation, respectively. It remains unclear why the French study showed much higher relapse rates [5]. One explanation might be the longer follow-up period or the higher amount of homozygous *MCP* mutations within the cohort of Fremeaux-Bacchi et al. (where 5 out of 12 patients had homozygous mutations) [5]. The number of homozygous mutations within their cohort could also explain the earlier age of onset compared with our own cohort (1.5 vs. 4.58 years) [5]. Additionally, different ethnic backgrounds could explain the variable clinical courses. The patients with relapses had a trend towards earlier onset of the disease compared with non-relapsing patients, but also a longer mean observation period. Therefore, additional studies with larger case numbers and longer follow-up periods are needed to further study the relapse rate of pediatric patients with *MCP* mutations.

The overall kidney outcome in our cohort was good with a median eGFR above 100 ml/min/1.73m^2^ at last follow-up. Only one patient developed CKD stage 5, 5.6 years after onset of aHUS. This is comparable with the published data demonstrating that *MCP* mutations cause milder kidney damage than other forms of genetic aHUS, but still can lead to kidney failure [35].

Currently, the recommended therapy for most aHUS patients is long-term administration of eculizumab, with a maintenance-dosing interval twice a month [3]. The efficacy of this treatment has been shown in several prospective clinical trials, performed initially in adults and later in children [11, 14, 37, 38]. However, discontinuation of eculizumab has been controversially discussed, as eculizumab has also potential adverse effects, especially with long-term treatment [15–18, 21, 39]. In our cohort, only 4 out of 20 patients received long-term and 3 out of 20 patients temporary eculizumab treatment, which is explained partly by the onset of aHUS before the era of this treatment option.

As long-term eculizumab therapy is expensive and can lead to complications, we feel the role of eculizumab long-term treatment in patients who may never develop kidney failure or relapses needs to be considered carefully. Considering the low overall risk of kidney failure and a relapse risk of 10% per year, we personally are in favor of short-term eculizumab treatment.

Many centers already choose not to follow the recommended long-term regimen, especially for patients with underlying *MCP* mutations and thus low risk of CKD stage 5. Wijnsma et al. have developed a treatment protocol of a restrictive eculizumab therapy for aHUS patients in the Netherlands [18]. They recommend that irrespective of the underlying mutation, all aHUS patients should receive eculizumab, but after 3 months treatment should be re-evaluated and the drug should either be withdrawn or tapered [18]. In our opinion, risks and benefits of long-term vs. short-term/on-demand eculizumab treatment have to be considered carefully in each individual case. While receiving eculizumab, the risk of meningococcal infection is increased more than 2000-fold compared with the healthy population [21, 40, 41]. As eculizumab has only been used for aHUS since 2009, long-term and rare adverse effects have only systematically been analyzed over a period of 5 years from the global aHUS registry [20]. After administration of eculizumab in the acute phase, it is difficult to motivate patients and parents in stable remission to undergo indefinite treatment without evidence of substantial benefit from prospective clinical trials. In our view, a short-term/on-demand eculizumab therapy, as proposed in the Netherlands, is probably sufficient for the majority of patients with *MCP* mutations. The pre-requisite for this approach has to be a well-educated and compliant patient and family who are able to dipstick the urine regularly at home and measure blood pressure. In case of any sign of a potential relapse (microhematuria, proteinuria, oliguria, or potentially aHUS-triggering infections), the patient will have to be seen by a pediatrician immediately and eculizumab should be applied in case of relapse under pediatric nephrology care. As suggested by Wijnsma et al., we propose a re-evaluation of the treatment regimen at earliest after 3 months [18]. In case of remission (normalized kidney function and hematological parameters), and depending on the amount of proteinuria, a withdrawal or tapering of eculizumab therapy should be taken into consideration. However, the current data on discontinuation of eculizumab is based on retrospective data from clinical case reports [13] and analyses, as summarized above*.* Also, we could not show a clear benefit from complement blockade therapy, independently of short- or long-term treatment. However, this may have been due to a small treatment group and high loss of follow-up in the non-relapsing group. Thus, independent prospective and larger observational studies or randomized controlled trials for pediatric and adult patients with isolated *MCP* mutations are needed. Currently, a prospective observational study (NCT02574403) for all aHUS patients (> 3 years of age) to define the outcome after treatment discontinuation has been completed and results are expected shortly. This will help to predict kidney outcome and identify the group of patients who would benefit from long-term vs. short-term eculizumab treatment. Furthermore, potential relapse triggers and diagnostic tools for early detection of relapses should be identified.

Limitations of our study are the retrospective survey-based approach and the variable observation periods including a large number of patients who were lost to follow-up, especially in the non-relapsing group. A potential explanation for this observation was that patients without relapsing disease rather tended to miss follow-up visits compared with those with relapse experience. Even though a longer follow-up would have improved data quality, the Kaplan-Meier survival analysis censors drop-out patients and shows the relapse likelihood.

In summary, our retrospective analysis showed that without preventive therapy, children with isolated heterozygous *MCP* mutations have about a 40% risk of first relapse over 5 years. With the limitation of small treatment groups, there was no significant difference in long-term kidney function in the groups with vs. without and long- vs. short-term eculizumab treatment. Nevertheless, in our opinion, it appears safe to discontinue initial eculizumab treatment in individual patients in this subgroup, with an immediate restart of eculizumab in case of relapse. We think that larger observational studies are urgently needed, especially for patients with *MCP* mutations, in order to minimize the risks of the disease and its therapy while optimizing the treatment benefits.

## Electronic supplementary material

ESM 1 (DOCX 25 kb)
