# Supplementary material for: Different approaches to long-term treatment of aHUS due to MCP mutations: a multicenter analysis
Source: Pediatr Nephrol. 2020 Jul 26;36(2):463–71. doi: 10.1007/s00467-020-04714-0 (PMC7815604; doi:10.1007/s00467-020-04714-0)
Supplement: Supplementary file 1 — (DOCX 25 kb) [file 467_2020_4714_MOESM1_ESM.docx]

**Suppl. Table 1. 20 pediatric patients with atypical hemolytic uremic syndrome and a monogenic mutation in the gene *MCP.***

| **Family Nr.** | **Gen-**  **der** | **Nucleotide change** | **Amino acid change** | **Exon (Zygo-sity)** | **Published in** | **Conservation** | **SIFT** | **MT** | **PP2** | **GnomAD (hom/het/all)** |
| --- | --- | --- | --- | --- | --- | --- | --- | --- | --- | --- |
| 1 | m | c.126_127del | p.Glu42Aspfs*7 | 2 (het) | novel | n/a | n/a | n/a | n/a | - |
| 2 | m | c.286+2T>G | p.? | Intron 2 (het) | 27 | n/a | n/a | n/a |  | 0/13/249756 |
| 3 | m | c.104G>A | p.Cys35Tyr | 2 (het) | 26 | *D. rerio* | Del | DC | 1 | 0/3/251344 |
| 4 | m | c.608T>G | p.Ile203Ser | 5 (het) | novel | *G. gallus* | Tol | DC | 0,902 | - |
| 5 | f | c.800_820del | p.Thr267_Asn273del | 6 (het) | novel | n/a | n/a | n/a | n/a | 0/3/251248 |
| 6 | m | c.104G>A | p.Cys35Tyr | 2 (het) | 26 | *D. rerio* | Del | DC | 1 | 0/3/251344 |
| 7 | f | c.623T>C | p.Ile208Thr | 5 (het) | novel | *G. gallus* | Tol | Poly | 0,988 | - |
| 8 | m | c.286+2T>G | p.? | Intron 2 (het) | 27 | n/a | n/a | n/a | n/a | 0/13/249756 |
| 9 | m | c.286+2T>G | p.? | Intron 2 (het) | 27 | n/a | n/a | n/a | n/a | 0/13/249756 |
| 10 | m | c.286+2T>G | p.? | Intron 2 (het) | 27 | n/a | n/a | n/a | n/a | 0/13/249756 |
| 11 | f | c.676G>T | p.Val226Phe | 6 (het) | novel | *X. tropicalis* | Tol | DC | 0,99 | - |
| 12 | f | c.175C>T | p.Arg59* | 2 (het) | 26 | n/a | n/a | n/a | n/a | 0/3/251414 |
| 13 | m | c.286+2T>G | p.? | Intron 2 (het | 27 | n/a | n/a | n/a | n/a | 0/13/249756 |
| 14 | m | c.485G>A | p.Cys162Tyr | 5 (het) | novel | *D. rerio* | Del | DC | 1 | - |
| 15 | m | c.175C>T | p.Arg59* | 2 (het) | 26 | n/a | n/a | n/a | n/a | 0/3/251414 |
| 16 | m | c.565T>G | p.Tyr189Asp | 5 (het) | 28 | *D. rerio* | Del | Poly | 1 | 0/5/282838 |
| 17 | m | c.685C>T | p.Arg229* | 6 (het) | 29 | n/a | n/a | n/a | n/a | - |
| 18 | m | c.286+2T>G | p.? | Intron 2 (het) | 27 | n/a | n/a | n/a | n/a | 0/13/249756 |
| 19 | f | c.485G>A | p.Cys162Tyr | 5 (het) | novel | *D. rerio* | Del | DC | 1 | - |
| 20 | m | c.380G>A | p.Cys127Tyr | 3 (het) | novel | *D. rerio* | Del | DC | 1 | - |

**Abbreviations:**  DC, disease causing; Del, deleterious; f, female; gnomAD, Genome Aggregation database; het, heterozygous; hom, homozygous; MT, mutation taster; m, male; n/a, not applicable; Nr, number; PP2, PolyPhen-2 prediction score; SIFT, “Sorting Tolerant from Intolerant” prediction score.
